# MEK Inhibitor Augments Antitumor Activity of B7-H3-Redirected Bispecific Antibody

**DOI:** 10.3389/fonc.2020.01527

**Published:** 2020-08-25

**Authors:** Hongjian Li, Cheng Huang, Zongliang Zhang, Yunyu Feng, Zeng Wang, Xin Tang, Kunhong Zhong, Yating Hu, Gang Guo, Liangxue Zhou, Wenhao Guo, Jianguo Xu, Hui Yang, Aiping Tong

**Affiliations:** ^1^State Key Laboratory of Biotherapy and Cancer Center, West China Hospital, Sichuan University and Collaborative Innovation Center for Biotherapy, Chengdu, China; ^2^Department of Neurosurgery, West China Medical School, West China Hospital, Sichuan University, Chengdu, China; ^3^Department of Abdominal Oncology, West China Medical School, West China Hospital, Sichuan University, Chengdu, China; ^4^Department of Otolaryngology, Head and Neck Surgery, West China Medical School, West China Hospital, Sichuan University, Chengdu, China

**Keywords:** trametinib, B7-H3, immunotherapy, bispecific antibody, non-small-cell lung cancer, bladder cancer

## Abstract

Targeting cancer antigens by T cell-engaging bispecific antibody (BiAb) or chimeric antigen receptor T cell therapy has achieved successes in hematological cancers, but attempts to use it to fight solid cancers have been disappointing, in part due to antigen escape. MEK inhibitor had limited activity as a single agent, but enhanced antitumor activity when combined with other therapies, such as targeted drugs or immunotherapy agents. This study aimed to analyze the expression of B7-H3 in non-small-cell lung cancer (NSCLC) and bladder cancer (BC) and to evaluate the combinatorial antitumor effect of B7-H3 × CD3 BiAb with MEK inhibitor trametinib. We found B7-H3 was highly expressed in NSCLC and BC compared with normal samples and its increased expression was associated with poor prognosis. Treatment with trametinib alone could induce apoptosis in tumor cell, while has no effect on T cell proliferation, and a noticeable elevation of B7-H3 expression in tumor cells was also observed following treatment. B7-H3 × CD3 BiAb specifically and efficiently redirected their cytotoxicity against B7-H3 overexpressing tumor cells both *in vitro* and in xenograft mouse models. While trametinib treatment alone affected tumor growth, the combined therapy increased T cell infiltration and significantly suppressed tumor growth. Together, these data suggest that combination therapy with B7-H3 × CD3 BiAb and MEK inhibitor may serve as a new therapeutic strategy in the future clinical practice for the treatment of NSCLC and BC.

## Introduction

Lung cancer is the second most common cancer with a 5-years survival rate of 19% (1). Non-small-cell lung cancer (NSCLC) accounts for 85% of all lung cancer diagnoses (2, 3). Bladder cancer (BC) is the ninth most common cancer worldwide, which is responsible for more than 160,000 deaths each year (1, 4). Although the progress in modern treatment modalities including surgical resection, chemotherapy, radiotherapy and targeted therapy, patients with NSCLC and BC still suffer from significant treatment failure due to high rates of recurrence and poor prognosis for advanced disease (5, 6). Thus, novel treatment regimens are urgently needed for NSCLC and BC.

B7-H3, a type I transmembrane protein, is a member of the B7 family with immune modulatory functions (7, 8). The expression of B7-H3 is absent or low in normal human tissues (9). Interestingly, it is frequently upregulated in a high proportion of human malignancies, such as head and neck cancer and medulloblastoma (9–12). As a result, B7-H3 is considered as a promising biomarker and target for cancer immunotherapy. A few B7-H3-directed CAR T cells have been undertaken in preclinic models (13–17). Besides, recent studies have suggested that B7-H3 promotes the migration and invasion of NSCLC and BC cells (12, 18), and overexpression of B7-H3 is usually related to a worse clinical prognosis (11, 19). Therefore, B7-H3 may be an attractive target for NSCLC and BC.

It has become a major breakthrough for tumor immunotherapy by engaging the immune system to eradicate tumor cells. In the forefront of these treatments, the most promising approach is T-cell-based therapies, including the T-cell-engaging bispecific antibody (BiAb). It can facilitate T cell recruitment and target cell killing by binding to the T-cell receptor CD3 subunit and tumor cells via a tumor-associated antigen (TAA) (20). Compared with CAR-T cell therapy, the strength of T-cell-engaging BiAb lies in the fact that it recruits endogenous T cells to tumors without the need to manipulate T cells *ex vivo* in a patient-specific manner (21, 22). So far, a few studies on T-cell-engaging BiAb have been reported for various cancer treatment (23–28). However, novel strategies are still needed to overcome antigen escape in solid tumors, which is a main drawback of BiAb (29).

Abnormal mitogen-activated protein kinase (MAPK) signaling is associated with the occurrence and development of various cancers (30). Aberrant activation of MAPK can be induced by a variety of mutations, such as RAS, RAF, and MEK1/2 (31). Notably, MEK1/2 mutations are common in several cancers, including lung cancer and bladder cancer (30, 32–34). Trametinib is an oral, reversible and highly selective inhibitor of MEK1/2 (34). Compared with other inhibitors, trametinib exhibits superior performance due to its favorable pharmacokinetics, long biological half-life, minor side effect and low risk of adverse drug reactions (31). Inhibition of oncogenic MAPK signaling by trametinib has been an effective strategy to treat metastatic melanoma (35). However, there are limitations for trametinib to fight against solid cancers, due to the acquisition of resistance after repeated administration (36). Thus, combination with trametinib and immunotherapy may be a promising therapeutic schedule.

Herein, to develop a new NSCLC and BC treatment modality, we tried to construct a B7-H3 × CD3 BiAb that binds to T cells and target surface expressed on tumor cells. In addition, we selected a MEK inhibitor trametinib for combination therapy. We hypothesized that the BiAb and trametinib could separately mitigate tumor cells' malignant phenotype. Furthermore, we sought to test whether trametinib would improve the bispecific antibody responses *in vitro* and *in vivo*.

## Materials and Methods

### Mice

Six-to-eight-weeks-old immunodeficient NOD-SCID female mice were purchased from the Model Animal Resource Information Platform of Nanjing University. Mice were maintained under specific pathogen-free facilities at Sichuan University. All procedures met the requirements of the National Institutes of Health and Institutional Animal Care and Use Committee.

### Tissue Microarray and Blood Samples

Human tissue microarrays for immunohistochemistry (IHC) were purchased from Xi'an Alenabio and Shanghai Outdo Biotech of China. Blood samples from healthy donors were used for isolation of human peripheral blood mononuclear cells (PBMCs).

### Cell Lines and Culture Conditions

Human NSCLC cells A549 (which has the KRASQ61H mutation), H460 (which has the KRASG12S mutation), BC cells T24 (which has the HRASG12V mutation), and HEK293T cell lines were purchased from ATCC. A549, H460, and HEK293T cell lines were maintained in Dulbecco's modified Eagle medium (Gibco) with 10% fetal bovine serum (Gibco) and 2 mmol/L L-glutamine. T24 cell line was maintained in McCoy's 5A Medium (Gibco) with 10% fetal bovine serum and 2 mmol/L L-glutamine.

PBMCs were isolated using density gradient centrifugation and activated by culturing with anti-CD3 mAb (OKT3, 100 ng/mL, BioLegend), anti-CD28 mAb (CD28.2, 100 ng/mL, Sino Biological), and recombinant human interleukin-2 (IL-2) (100 units/mL, Life Science) in X-Vivo medium (Lonza) supplemented with 10% fetal bovine serum (heat inactivation at 56°C for 30 min) and 2 mM L-glutamine and for 3 days.

### Construction and Production of B7-H3 × CD3 BiAb

The BiAb was constructed by our previous description (37). Briefly, the anti-B7-H3 single-chain variable fragment (scFv) sequence was derived from a highly specific monoclonal antibody against B7-H3 (clone mAb-J42) generated by our group using a standard hybridoma technique. cDNAs encoding the anti-B7-H3 scFv and anti-CD3 scFv (according to published amino acid sequences) were synthesized by commercial gene synthesis service (Genewiz). The two scFvs were linked by a G4S linker to construct a recombinant single-chain BiAb. The cDNAs were subcloned into an expression vector with a His tag at the C-terminal for protein purification.

HEK293T cells were transfected with the vector described above and cultured in the FreeStyle serum-free medium (Thermo Fisher Scientific) at 37°C, 5% CO_2_. After 7 days, culture supernatant was harvested and pre-cleaned by 0.45 μm filters. The BiAb was purified on Ni-NTA affinity columns and subsequently subjected to size exclusion chromatography. To assess the molecular mass of the BiAb, obtained samples were subjected to SDS-PAGE and stained with Coomassie brilliant blue.

### Immunofluorescence Staining

Cells were incubated in 24-well plates under standard cell culture conditions (5 × 10^3^ cells per well). After 12 h, cells were blocked with 5% BSA for 15 min, stained with B7-H3 antibody (Abcam, ab227679) for 1 h, Cy3-conjugated secondary antibody (Beyotime, A0516) for 40 min and DAPI (Beyotime) in the dark. Images were captured on a fluorescence microscopy.

Tumor tissues from the T cell group mice were collected and immediately froze at −80°C. Sections were fixed in pre-chilled acetone-methanol (1:1) for 20 min at −20°C and then allowed to air-dry for 10 min before being blocked with 5% BSA for 30 min. Subsequently, sections were stained with B7-H3 antibody (Abcam, ab227679) for 1 h, FITC-conjugated secondary antibody (Beyotime, A0562) for 40 min and DAPI (Beyotime) in the dark. Images were captured on a fluorescence microscopy.

### Flow Cytometry

B7-H3 expression level on tumor cells was analyzed by flow cytometry. Cells were collected by centrifugation and incubated with the human B7-H3 antibody (BioLegend, 331605) in 500 μL PBS for 20 min in the dark. After washing three times with PBS, the cells were resuspended in 500 μL PBS and analyzed using a NovoCyte™ Flow Cytometer (ACEA Bioscience) according to the manufacturer's protocols. For T cell phenotype analyses, human CD4 (BioLegend, 357419), CD8 (BioLegend, 344729), CD25 (BioLegend, 302629), and CD69 (BioLegend, 310909) antibodies were used and experiments were performed on a Fortessa flow cytometer (BD).

For apoptosis detection, Annexin V staining was measured by FITC-annexin-V Apoptosis Detection Kit I (4A Biotech). A549 and H460 cells (5 × 10^5^) were treated for 48 h with 10 μM trametinib (MCE). Cells were collected and resuspended in 1 × Binding Buffer, 100 μL solution (1 × 10^5^ cells) was used to stain cells with 5 μL FITC annexin V for 15 min in the dark followed by the addition of 0.4 mL of 1 × Binding Buffer and 10 μL 7-AAD. Flow cytometry analysis was performed on a NovoCyte™ Flow Cytometer (ACEA Bioscience) according to the manufacturer's protocols.

For T cell proliferation assay, T cells were initially stained with carboxy fluorescein succinimidyl ester (CFSE) (Beyotime) and cultured in the presence or absence of 1 μM trametinib. After 48 h, cell proliferation was carried out by flow cytometry.

### Western Blotting

Cells after treatment with the indicated concentrations of trametinib for 48 h were lysed in RIPA buffer (Beyotime), supplemented with protease and phosphatase inhibitors (sigma). All procedures were conducted on ice. Total proteins were extracted from cells and quantified by BCA protein assay kit (Beyotime). Then, equal amount of proteins (10 μg) was subjected to SDS-PAGE and transferred to polyvinylidene difluoride membranes. After that, the membranes were blocked with 5% milk for 1 h. Subsequently, the membranes were stained with different primary antibodies, including B7-H3 (CST, 14058S), MEK1/2 (CST, 8727T), P-MEK1/2 (CST, 3958S), and β-actin (ZSGB-BIO, TA09) antibodies for 1 h, HRP-conjugated secondary antibody (Beyotime, A0208) for 1 h. Images were captured by a ChemiScope 6000 Touch (Clinx).

### Cell Viability Assay

Cells were seeded in 96-well plates and incubated overnight prior to treatment. After 48 h with the indicated concentrations of trametinib, 10 μL of the Cell Counting Kit-8 solution (Beyotime) was added to each well and incubated for 2 h in the dark. Absorbance at 450 nm was measured in a microplate reader.

### Cytotoxicity Assays

A 2D and 3D co-culture models of tumor cells with human T cells were used to assess the cytotoxicity. In the 2D co-culture model, A549, H460, or T24 cells were co-cultured with T cells at an E:T ratio of 1:4, 1:1, and 4:1, together with 1 μM trametinib alone or in combination with 5 μg/mL BiAb. Images were captured at 12 and 24 h. To assess the effect of cytotoxicity, Chromium-51 assay was carried out as described (14). Tumor cells were labeled with sodium chromate (molecular formula, ${\text{Na}}_{2}^{51}$CrO_4_) and incubated with T cells at an E:T ratio of 1:4, 1:1, and 4:1 for 4 h. Then the radioactivity of the supernatants was measured by a gamma counter. The percentage of specific lysis was calculated by the formula: (test release-spontaneous release)/(maximal release-spontaneous release) × 100.

To further evaluate the cytotoxicity through combination therapy in 2D models, we utilized the xCELLigence real-time cells analyzer (ACEA Biosciences, Inc.). Briefly, H460 cells were seeded in the E-plate 96 well at 8 × 10^3^ cells per weel. After 15 h, samples were cultured with T cells at the E:T ratio of 4:1 and divided into four groups. Then cells were treated with trametinib (1 μM), the BiAb (5 μg/mL), or mock treatment with equal amount of DMSO. The combination groups received both trametinib and the BiAb at the above doses. Thereafter, the impedance was continuously measured for 60 h. Cell index, correlated with the cell viability and/or cytotoxicity, was automatically calculated from the impedance.

For the 3D spheroid model, the method was performed according to our previous description (38). In brief, 1 × 10^5^ cells were added to the Matrigel-coated wells and cultured in serum-free DMEM (Gibco) with 2% B-27 supplement (Gibco), 20 ng/ml human EGF (Sino Biological), and 20 ng/ml human bFGF (Sino Biological). After 5 days, human T cells were stained with CFSE (Beyotime) and added to the wells at the E:T ratio of 1:1, together with 5 μg/mL BiAb alone or in combination with 1 μM trametinib for 12 h. Cells were stained with DAPI (Beyotime) in the dark and images were captured on a fluorescence microscopy.

### Analysis of Cytokine Secretion

Tumor cells were co-cultured with T cells alone or together with 5 μg/ml BiAb in 24-well plates at different E:T ratio with. After 24 h, the supernatant was collected to evaluate the IFN-γ secretion by ELISA kits (BioLegend) according to the manufacturer's protocols.

### *In vivo* Experiments

In the H460 and T24 xenograft experiments, 2 × 10^6^ H460 or T24 cells were subcutaneously injected into NOD-SCID mice and were randomly divided into four groups consisted of *n* = 5 per group. From the tenth day on, trametinib (0.6 mg/kg) or vehicle control was administered for 10 consecutive days via oral gavage. On day 13, all mice were intravenously treated with 8 × 10^6^ T cells and from the day on, mice were intravenously treated with 100U IL-2 or in combination with 2 mg/kg BiAb or PBS for 7 consecutive days. The mice in the combination treatment group received both trametinib and the BiAb at the above doses and schedule. The vehicle control of trametinib was a mixture of 30% PEG400, 0.5% Tween80, and 5% propylene glycol. Bodyweight and tumor sizes were measured every 3 days. The tumor volume was calculated using the following equation: (length × width × width)/2.

### IHC Assay

Tumor, heart, liver, spleen, lung, and kidney sections from mice were preprocessed by paraformaldehyde and embedded in paraffin. After slicing into sections, slides were performed with H&E staining. Tumor paraffin sections were immunostained with CD3 (Servicebio, GB13014), CD31 (Servicebio, GB11063), or caspase-3 (Servicebio, GB11009) antibody. All procedures followed the manufacturer's protocol. In brief, tissue sections were incubated at 65°C for 1 h to retrieve antigenicity, blocked with PBS containing 10% normal goat serum for 30 min at room temperature, and then incubated with primary antibody at 4°C overnight. The sections were then incubated with secondary antibodies, and the staining was detected with 3,3′ diaminobenzidine (ZSGB-Bio).

### Statistical Analysis

Data were presented as the mean ± SD. Statistical analyses were performed using GraphPad Prism 7.0. The difference between various experimental and control groups was examined by Student's *t*-test and considered significant at ^*^P < 0.05; ^**^P < 0.01; ^***^P < 0.001. For bioinformatic analysis of B7-H3, the relationship between B7-H3 expression and prognosis was performed using the dataset of the Kaplan-Meier Plotter (KM Plotter) (39). The meta-analysis and mRNA expression of B7-H3 in tumor and normal tissues was analyzed by using the Oncomine (40). The association of B7-H3 expression and the tumor stage were examined by data mining in Oncomine or The Cancer Genome Atlas (TCGA). Gene expression analysis was performed together with the computation of the associated box plots and violin plots in R (Version 3.6.1) using the ggpubr package (Version 0.2.4) [https://CRAN.R-project.org/package=ggpubr] and the ggstatsplot [https://CRAN.R-project.org/package=ggstatsplot].

## Results

### Analysis of B7-H3 Expression and Survival From Online Database and Samples

**Figure 1:**
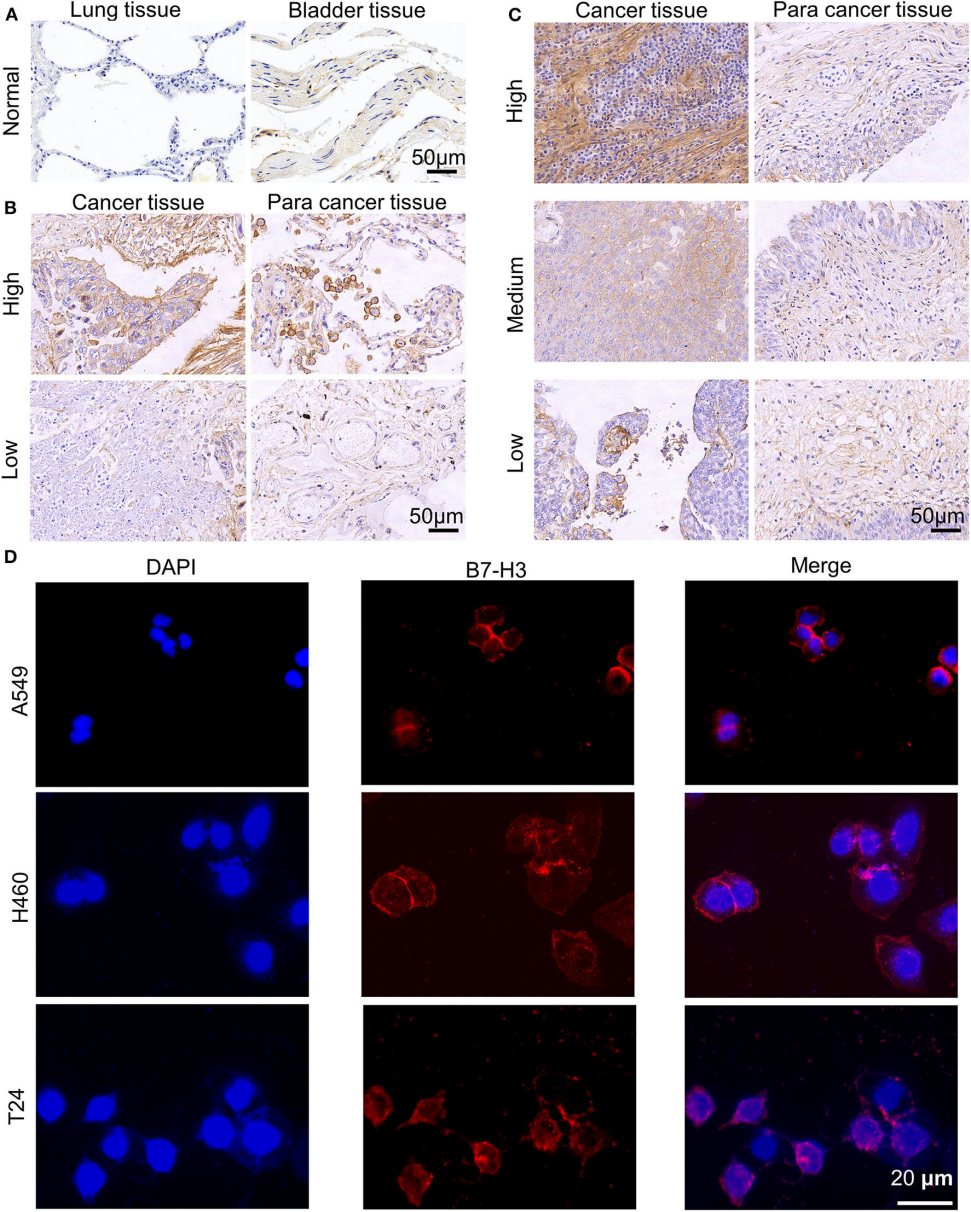
Expression of B7-H3 on human NSCLC and BC. **(A–C)** B7-H3 IHC staining patterns. Representative cases of normal lung and bladder samples **(A)**, NSCLC **(B)**, and BC **(C)** samples including para cancer tissues at different grades (high, medium or low). Scale bar, 50 μm. **(D)** Immunofluorescence staining of A549, H460, T24 tumor cells for B7-H3. Scale bar, 20 μm.

Based on the data, we analyzed the association of B7-H3 expression with survival in 1145 NSCLC and 404 BC patients (Supplementary Figures 1A,B). As shown, higher expression of B7-H3 was significantly correlated to lower survival in both NSCLC (*P* < 0.001) and BC (*P* < 0.001). Then, we tested the presence of B7-H3 in major subtypes of NSCLC and BC. We found differential B7-H3 expression with significantly higher levels in NSCLC and BC subtypes as compared with normal lung and bladder samples (*P* < 0.05; Supplementary Figures 1C,D). In addition, the association between B7-H3 expression and clinical stage of NSCLC or BC patients was also evaluated. There was no significant difference in the expression of B7-H3 (*P* = 0.748; Supplementary Figure 1E) in NSCLC patients with different pathologic stages. However, advanced stage BC patients were likely to show higher B7-H3 expression compared with early stage patients (*P* = 0.002; Supplementary Figure 1F). Also, the results in heat maps of the previous studies (41–46) further implied B7-H3 was highly expressed in NSCLC and BC samples compared to normal tissues (Supplementary Figure 2). After that, we performed IHC staining to identify the expression of B7-H3 in tissue microarrays, including tumor, tumor-adjacent, and normal tissues at different grades (Figures 1A–C). The intensity of B7-H3 expression was markedly increased in malignant tumor and tumor-adjacent tissues compared to normal tissues. Finally, we evaluated the expression of B7-H3 in A549, H460, and T24 cell lines using immunofluorescence and flow cytometry. As shown, B7-H3 stained positively in NSCLC and BC cell lines (Figure 1D). Together, these results indicated that the B7-H3 marker might serve as a clinical target for the treatment of patients with NSCLC and BC.

### MEK Inhibitor Trametinib Suppressed Cell Growth and Increased the Expression of B7-H3 in NSCLC and BC Cell Lines

**Figure 2:**
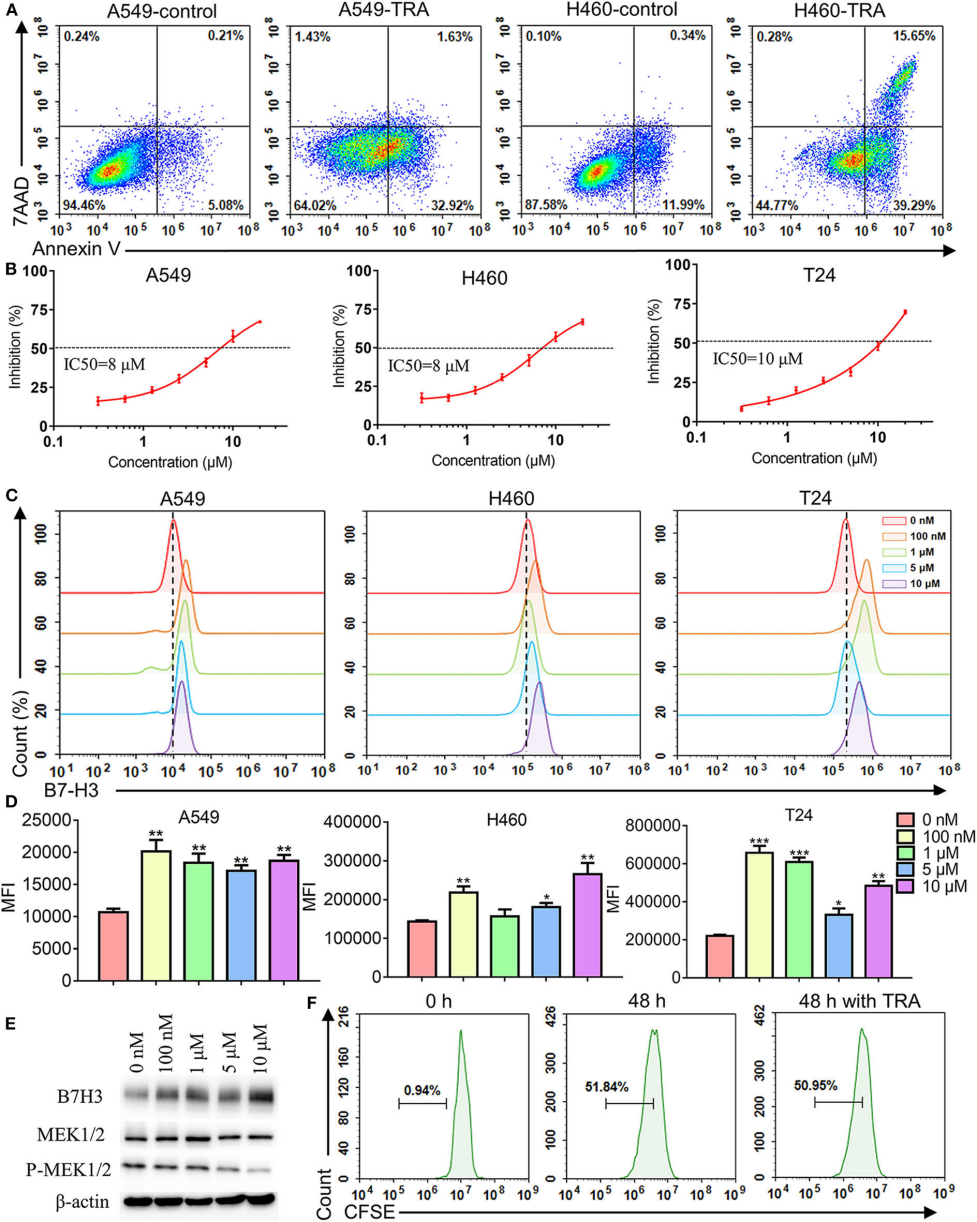
Effects of MEK inhibitor trametinib in A549, H460, T24, and human T cells. **(A)** Apoptosis detection with annexin V-FITC/7AAD double staining by flow cytometry. A549 and H460 cells were cultured with 10 μM trametinib for 48 h. **(B)** Inhibition rate of A549, H460, and T24 cells after exposure to trametinib with various concentrations. After 48 h of treatment, inhibition rate was measured using Cell Counting Kit-8 assays. **(C)** B7-H3 expression after trametinib treatment with indicated concentrations by flow cytometry. Histograms represent the measured fluorescence of cells incubated with the B7-H3 antibody. **(D)** Histogram of the mean fluorescence intensity. **(E)** Western blot analysis of B7-H3, MEK, and P-MEK expression in A549 cells after trametinib treatment with indicated concentrations. Expression of β-actin was used as an internal control. **(F)** Proliferation (CFSE dilution assay) of human T cells after 48 h of treatment with 1 μM trametinib by flow cytometry. Histograms represent the measured fluorescence of cells incubated with CFSE. *P < 0.05, **P < 0.01, ***P < 0.001.

To explore the effect of MEK inhibitor trametinib on NSCLC and BC cell lines, we used flow cytometry and CCK-8 assay to investigate cell apoptosis and proliferation, respectively. The results of flow cytometry revealed that incubating A549, H460, and T24 with trametinib induced cell apoptosis compared with the control groups (Figure 2A, Supplementary Figure 3). Similarly, cells were treated with various concentrations of trametinib and the results showed that trametinib has a dose-dependent killing effect on A549, H460, and T24 cells, identified via CCK8 assay (Figure 2B). As shown, the inhibition did not exceed 25% when drug concentration was under 2 μM. However, when the concentration rose to 10 μM or higher, inhibition could be observed in more than 50% of the cells. The IC50 of trametinib on A549, H460, or T24 cells was 8, 8, or 10 μM, respectively. Furthermore, to investigate whether trametinib affected B7-H3 expression in cell lines, A549, H460, and T24 cells were cultured with different concentrations of trametinib for 48 h and the expression levels were determined by flow cytometry. Compared to control groups, we found that B7-H3 expression was significantly upregulated in A549, H460, and T24 cells after trametinib stimulation (Figure 2C). Based on the data, we analyzed the changes in percentage among various groups. The up-regulation rate of the 0 nM group was set at 0–1%. For A549 cell line, the percentage rose to 6–15% in 100 nM, 1, 5, and 10 μM groups. For H460 cell line, the percentage rose to 5–19% in 100 nM, 5 and 10 μM groups. For T24 cell line, the percentage rose to 11–25% in 100 nM, 1, and 10 μM groups. Data in Figure 2D showed the median fluorescence intensity (MFI) of flow cytometry. For A549 cell line, the MFI of control group was 10699 compared to 18597 after stimulation with trametinib. For H460 cell line, the MFI of control group was 143574 compared to 205619 after stimulation with trametinib. For T24 cell line, the MFI of control group was 220409 compared to 520226 after stimulation with trametinib. The elevation of B7-H3 expression was confirmed by western blot (Figure 2E). Treatment with trametinib also inhibit MEK signaling, based on lower levels of phosphorylated MEK (Figure 2E). In addition, we did not find that trametinib suppressed the proliferation of human T cells by CFSE staining (Figure 2F).

### Generation and Characterization of B7-H3 × CD3 BiAb

**Figure 3:**
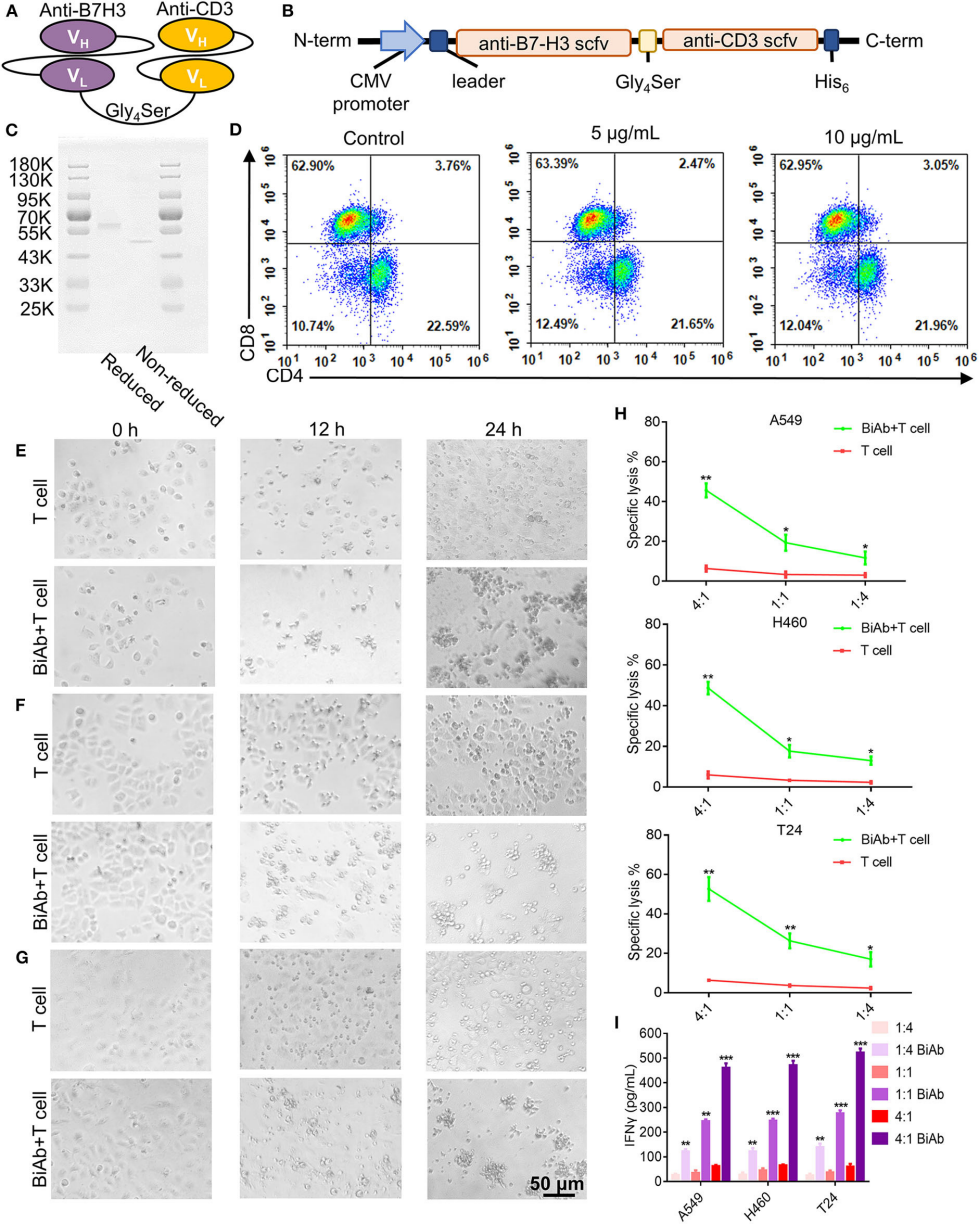
Construction, characterization and cytotoxicity of B7-H3 × CD3 BiAb. **(A)** The schematic representation of B7-H3 × CD3 BiAb. **(B)** Schematic diagram of B7-H3 × CD3 BiAb expression vector. **(C)** SDS-PAGE analysis of B7-H3 × CD3 BiAb. The BiAb was run on reducing and non-reducing SDS-PAGE gels. **(D)** Dot plot diagram of flow cytometry showing CD4^+^ and CD8^+^ percentage of human T cells after 5 or 10 μg/mL B7-H3 × CD3 BiAb treatment for 48 h. **(E–G)** Morphology of tumor cells after co-culture with human T cells. A549 **(E)**, H460 **(F)**, or T24 **(G)** cell lines were co-cultured with T cells for 12 or 24 h at a ratio of E:T = 4:1. Group “BiAb + T cell” was treated with B7-H3 × CD3 BiAb at a concentration of 5 μg/mL. Scale bar, 50 μm. **(H)** 51Cr-release assays of T cells against A549, H460 and T24 cell lines with 5 μg/mL B7-H3 × CD3 BiAb in different E:T ratios. **(I)** Quantification of IFN-γ by ELISA in the supernatant 24 h after co-culture of T cells with A549, H460, or T24 cell lines at different E:T ratio. Group “BiAb” was treated with B7-H3 × CD3 BiAb at a concentration of 5 μg/mL. *P < 0.05, **P < 0.01, ***P < 0.001.

B7-H3 × CD3 BiAb was engineered by combining a B7-H3 single chain variable region (scFv) with a CD3 scFv. Each scFv contained a corresponding light chain (VL) and heavy chain (VH) joined together by a 5-amino-acid (G4S) linker (Figures 3A,B). Figure 3C shows the SDS-PAGE analysis of purified B7-H3 × CD3 BiAb. Before the *in vitro* and *in vivo* antitumor assay, the ratio of CD4^+^/CD8^+^ human T cells stimulated by B7-H3 × CD3 BiAb was analyzed by flow cytometry (Figure 3D). Two days after stimulation, there was no significant difference between the BiAb stimulated T cells and control group.

### Functional Test of B7-H3 × CD3 BiAb *in vitro*

We photographed the growth of A549, H460, and T24 cells after 12- or 24-h incubation periods in the co-culture assay. Clusters of T cells and the lysis of cancer cells were observed in groups with the BiAb (Figures 3E–G, Supplementary Figure 4). We evaluated the cytotoxicity of the BiAb toward cancer cell lines by ^51^Cr-release assay and the results under different E/T ratios are shown in Figure 3H. After being treated with the BiAb, nearly 20% of the cells were lysed at an E/T ratio of 1:4. When the E/T ratio rose to 1:1, specific lysis was 20–30%. Interestingly, specific lysis rose to 40–60% at an E/T ratio of 4:1. Furthermore, co-culture assay was carried out and supernatants were collected to determine the relative cytokine secretion level. A significant increase in IFN-γ release can be detected in groups with the BiAb (Figure 3I).

### Cytotoxicity of B7-H3 × CD3 BiAb in Combination With Trametinib Is Superior to Single Agents *in vitro*

**Figure 4:**
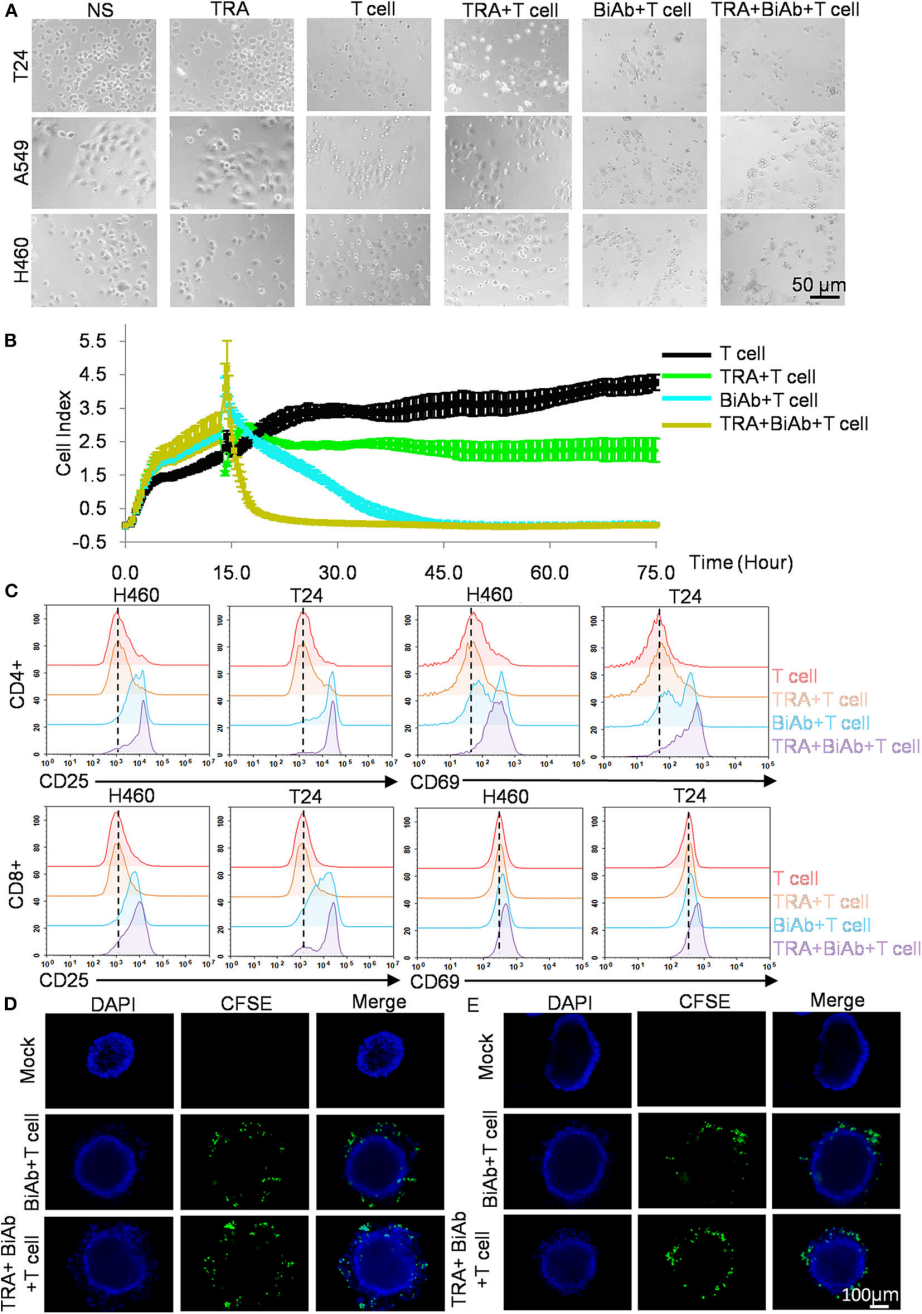
Antitumor activity by B7-H3 × CD3 BiAb in combination with trametinib *in vitro*. **(A)** Morphology of tumor cells after various treatments for 12 h. Scare bar, 50 μm. **(B)** Real-time cytotoxicity assay of H460 cells after various treatments for 75 h. **(C)** Activation signal of human T cells after co-culture with tumor cells by adding 1 μM trametinib alone or in combination with 5 μg/mL B7-H3 × CD3 BiAb. Cells were collected 24 h after co-culture with H460 and T24 cell lines and stained with antibodies against CD4, CD8, CD25, CD69 for flow cytometry. **(D,E)** Killing activity of B7-H3 × CD3 BiAb or in combination with trametinib was detected using the 3D tumorsphere model. A549 **(D)** and H460 **(E)** tumorspheres were co-cultured with CFSE labeled T cells including 5 μg/mL B7-H3 × CD3 BiAb alone or in combination with 1 μM trametinib for 12 h. Scare bar, 100 μm.

We sought to investigate whether combining B7-H3 × CD3 BiAb and trametinib can enhance tumor cell killing in NSCLC and BC cell lines. Co-culture assay was performed and representative bright-field images were shown in Figure 4A. Then, we tested the killing activity of various treatment groups on H460 cell line by real-time cytotoxicity assay (Figure 4B). As shown in the figure, the BiAb + T cell group showed a stronger inhibition effect compared to that of the TRA group. The combination group exhibited the best therapeutic results among all groups, suggesting the advantages of combination therapy. Next, T cells in co-culture assay were collected and the expression levels of CD25 and CD69 were detected to access T-cell activation (Figure 4C). Based on the data, we analyzed the changes in percentage among various groups. The positive rate of the T cell group was set at 3–6%. For CD25 analysis, the percentage rose to 22–52% in BiAb + T cell group and 45–64% in TRA + BiAb + T cell group. For CD69 analysis, the percentage rose to 10–37% in BiAb + T cell group and 26–54% in TRA + BiAb + T cell group. Then we compared the MFI among various groups. For the expression of CD25, the MFI of T cell and TRA + T cell group was 2803 compared to 12518 in BiAb + T cell group and 16698 in TRA + BiAb + T cell group. For the expression of CD69, the MFI of T cell and TRA + T cell group was 211 compared to 311 in BiAb + T cell group and 478 in TRA + BiAb + T cell group. These data showed that T cells, together with the BiAb alone or in combination with trametinib, exhibited higher degrees of CD25 and CD69 activation compared to control and trametinib alone groups. The ratio of CD4 and CD8 positive T cells was shown in Supplementary Figure 5. The proportion of CD4^+^/CD8^+^ T cells was not significantly altered throughout the co-culture assay. Furthermore, in the three-dimensional (3D) cancer spheroid model, although both groups with T cells and the BiAb were found to target tumorsphere, groups with trametinib were more lethal to spheres than groups without it (Figures 4D,E).

### Trametinib Enhanced the Anti-tumor Efficiency of B7-H3 × CD3 BiAb in Xenograft Models

**Figure 5:**
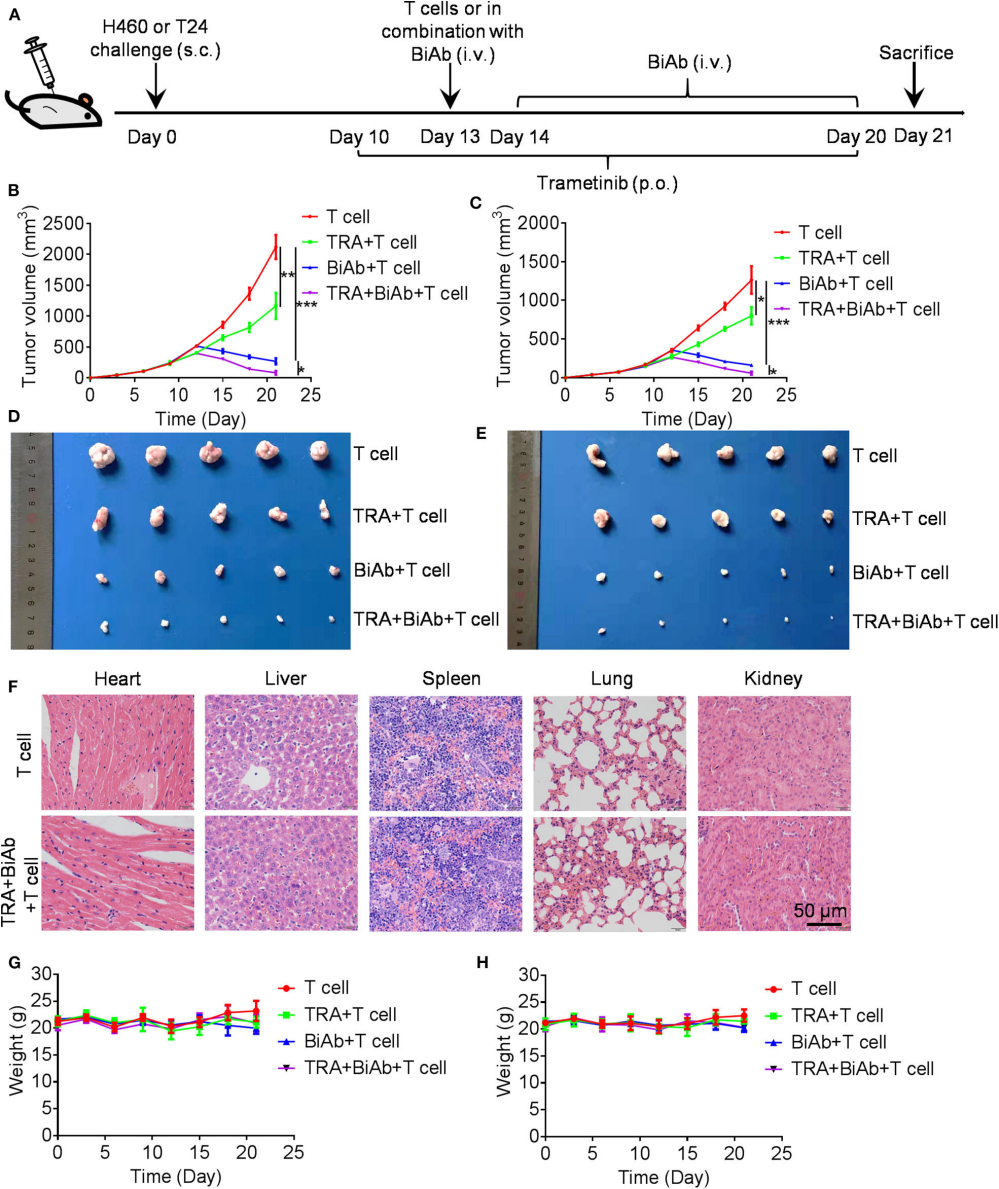
Antitumor activity by B7-H3 × CD3 BiAb in combination with trametinib *in vivo*. **(A)** Experiment design scheme. **(B,C)** Tumor growth curves from H460 **(B)** and T24 **(C)** mice models treated with T cell, T cell with trametinib, the BiAb or the BiAb combined with trametinib. Tumor volume measurements were recorded every 3 days. **(D,E)** Tumors from H460 **(D)** and T24 **(E)** mice models on day 21 are shown. **(F)** H&E staining images of liver, spleen, kidney, heart, and lung in H460 mice models. **(G,H)** Bodyweight of H460 **(G)** and T24 **(H)** mice treated with T cell, T cell with trametinib, the BiAb or the BiAb combined with trametinib. Bodyweight measurements were recorded every 3 days.

**Figure 6:**
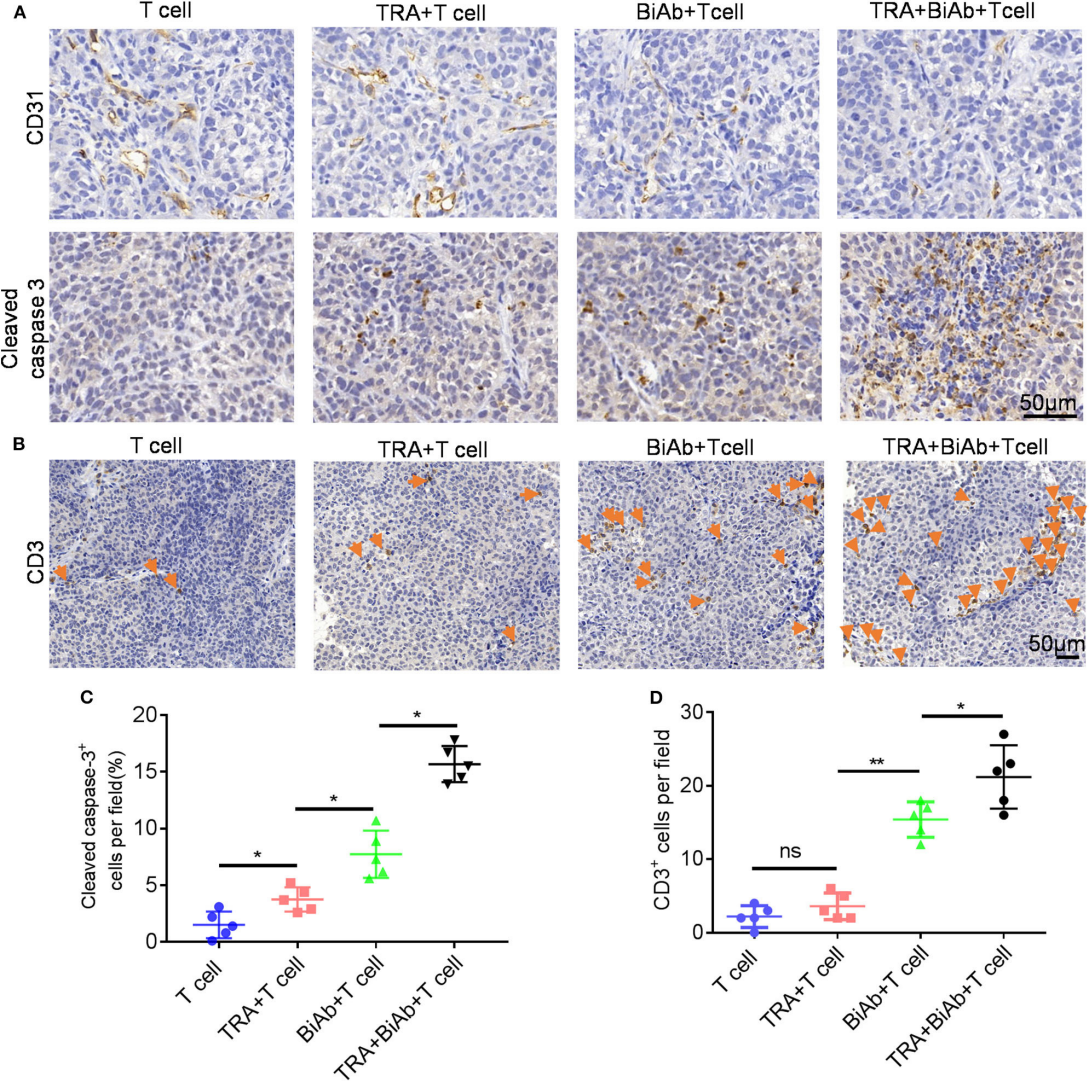
IHC analysis of tumors in H460 mice models. **(A)** Representative images of CD31 and caspase-3 staining in different groups. Scare bar, 50 μm. **(B)** Representative images of tumor-infiltrating T cells in different groups. T cells were detected by CD3 staining. Scare bar, 50 μm. **(C)** Quantification of cleaved caspase 3 in different groups. **(D)** Quantification of T-cell infiltration in different groups. *P < 0.05, **P < 0.01.

**Figure 7:**
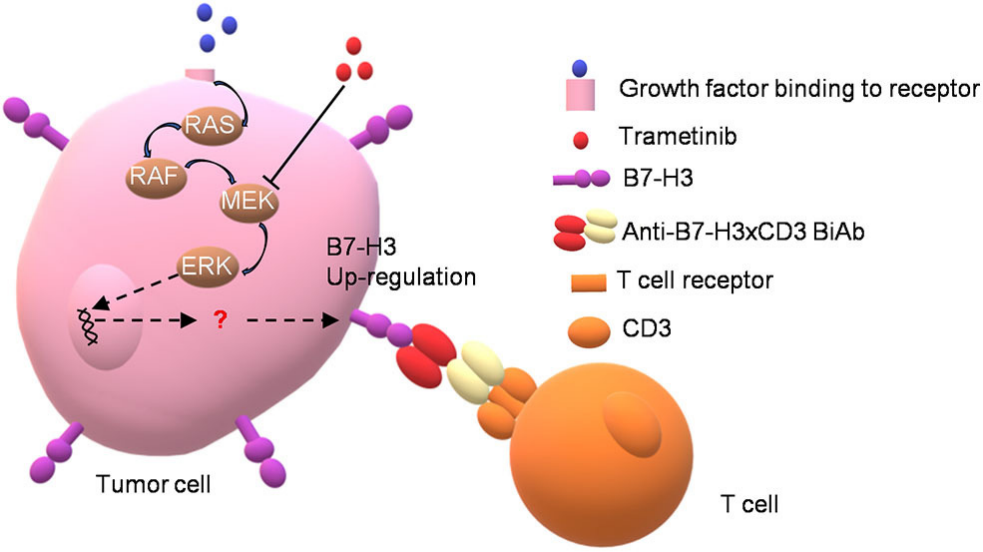
Schematic summary for synergistic effect between MEK inhibitor trametinib and B7-H3 × CD3 BiAb in killing NSCLC and BC cells.

To determine the *in vivo* efficacy of B7-H3 × CD3 BiAb and trametinib, NSCLC cell line H460 and BC cell line T24 were used in mice xenograft models. The schema is presented in Figure 5A. After subcutaneously injected with H460 or T24 cells, mice were daily administered with trametinib starting on day 10. Then mice were treated with identical doses of T cells, T cells with trametinib, T cells with the BiAb or T cells with the BiAb and trametinib at indicated time points. In the present study, NSCLC and BC tumor mass growth were significantly suppressed by trametinib, the BiAb or the BiAb combined with trametinib (Figures 5B,C). On day 21, although trametinib or the BiAb was able to inhibit tumor growth, the combination group was significantly more effective (Figures 5D,E). During the experiment, no abnormalities were observed in vital organs via H&E staining (Figure 5F). Also, there was no significant bodyweight loss in all groups (Figures 5G,H). We then analyzed mice-bearing tumor tissues by CD31 and caspase-3 immunostaining. The results showed that the combination groups appeared to have the highest rate of apoptosis and the lowest vessel density (Figure 6A). To further examine the recruitment of T cells in tumors, cell surface marker CD3 was analyzed. CD3^+^ T cells were seen in all groups (Figure 6B). Among these groups, the combined treatment group exhibited the highest apoptosis rate and number of CD3^+^ TILs, followed by the group treated with the BiAb and T cells (Figures 6C,D). The results of the study are summarized in a model diagram (Figure 7).

## Discussion

To date, the FDA has approved the combination of dabrafenib and trametinib for melanoma and lung cancer with the V600E BRAF mutation. Although response rates are ~60%, the incidence of adverse events is high and nearly all patients had at least one adverse event (98%) according to a phase II study (47). Besides, the durability of response is limited to 7–11 months due to drug resistance, which is a weakness of targeted therapy (48). By comparison, most immunotherapies do not deliver the same response rates but can offer higher durability (48). Thus, combination with trametinib and immunotherapy is attractive and meaningful. According to our data, overexpression of B7-H3 was revealed and it was associated with poor survival in NSCLC and BC. Both B7-H3 × CD3 BiAb and trametinib were able to inhibit NSCLC and BC cell growth *in vitro* and *in vivo*. A combination of trametinib and B7-H3 × CD3 BiAb inhibited NSCLC and BC cell growth and killed them by activating T cell *in vitro* as well as promoting T cell infiltration *in vivo*. In past studies, the inhibitory effect of trametinib on KRAS or BRAF mutant cell line has been confirmed (34, 49). Our data in A549 and H460 cell lines again corroborates the above results. It should be noted that we discovered trametinib could suppress the growth of T24 cells with HRAS mutation. Similarly, MEK inhibitor might be partly responsible for HRAS-mutated tumor reduction according to a phase I trial (50). This hint HRAS mutation cells may sensitize toward treatment with MEK inhibitors.

A previous report described the potential immunosuppressive activity of MEK inhibitors *in vitro*, which has limited the assessment of MEK inhibitor combination with immunotherapies (51). However, our data of CFSE labeling experiments did not show that trametinib could inhibit the proliferation of T cells. The difference in results is probably due to the fact that T cells were activated before adding trametinib in our experiments compared to the previous study that activated T cells during trametinib treatment. A recent research has implied that trametinib selectively blocked activation of naive T cells but did not suppress T cells which were already activated *in vitro* (52). Their results are in line with ours. Another study has demonstrated that MEK inhibitors potentiated rather than hindered antitumor T cells by impairing TCR-driven apoptosis (53). Similarly, we showed trametinib enhanced T cell activation in the co-culture assay. In addition, several studies have combined MEK inhibitor with PDL1 antibody or oncolytic virus and obtained ideal results (53–55). Taken together, these results suggest that blockage of MAPK signaling is critical and effective to prime and synergize tumors in response to immunotherapy.

It is essential to understand the effects of targeted agents on antitumor immune response. MEK inhibitors have been found to up- or down-regulate the expression of immune molecules, including MHC class I, PD-L1 in previous studies (52, 55–57). These contradictory results indicate that the differential expression of immune molecules may be context-dependent. Notably, a recent study has identified that there was a significant up-regulation of B7-H3 in the trametinib-treated A375 melanoma cell line (52). The present study had similar findings. We noticed that MEK inhibitor trametinib increased B7-H3 expression in human NSCLC and BC cell lines. This may be attributed to the complex regulatory pathways of B7-H3 which remains largely unknown. According to previous studies, B7-H3 can promote cell invasion via the STAT3 signaling pathway (58, 59). Meanwhile, trametinib was reported to upregulate MHC class I and PD-L1 by inducing STAT3 activation (55). However, whether trametinib regulates B7-H3 via the STAT3 or other signaling pathways requires further investigation. From our data, we can only infer it is feasible for combination therapy with trametinib and agents targeting B7-H3. Additional research is definitely needed to clarify the mechanism underlying our findings.

The most important factor in immunotherapy is to select a proper TAA to target. B7-H3 has been identified as promising immunotherapeutic targets for anticancer therapy, as it is aberrantly upregulated on the cell surface of many types of tumors (9–12). Several B7-H3 monoclonal antibodies have been tested in patients with refractory neoplasms (MGA271, clone 84D) (60) and glioma (antibody-drug conjugate, clone 8H9) (61). Recently, a B7-H3 × CD3 bispecific molecule (MGD009) is in clinical trials for solid tumors (ClinicalTrials.gov: NCT02628535). In our research, B7-H3 is confirmed abundant in NSCLC, BC cell lines and tissues, but it is expressed at low levels or almost undetectable in normal tissues. Moreover, according to our data, the therapeutic efficacy of B7-H3 × CD3 BiAb was significant and there were no related acute side effects. These results give further evidence that B7-H3 can act as an effective therapeutic target for the clinical management of NSCLC and BC.

In summary, we revealed that trametinib could inhibit cell proliferation and upregulate the expression of B7-H3 in tumor cells. B7-H3 × CD3 BiAb was able to directly guide T cell to kill tumor cells in human NSCLC and BC models. Moreover, we found trametinib could augment antitumor activity of B7-H3 × CD3 BiAb *in vitro* and *in vivo*. Although the molecular mechanism underlying the combination treatment needs to be further elucidated, these data provide new insights into NSCLC and BC treatment using a combination with MEK inhibitor and B7-H3-redirected immunotherapy.

## Data Availability Statement

Publicly available datasets were analyzed in this study. This data can be found here: Oncomine (www.oncomine.org); TCGA (http://cancergenome.nih.gov).

## Ethics Statement

The studies involving human participants were reviewed and approved by West China Hospital of Sichuan University Biomedical Ethics Committee (Ethical approval document: 2018-061). The patients/participants provided their written informed consent to participate in this study. The animal study was reviewed and approved by West China Hospital of Sichuan University Biomedical Ethics Committee (Ethical approval document: 2018-061).

## Author Contributions

AT, HL, CH, and HY designed the study. YF analyzed the data from Oncomine and TCGA. HL, CH, ZZ, ZW, GG, WG, and LZ performed the experiment. HL, ZZ, YF, ZW, XT, KZ, YH, and JX wrote the manuscript. All authors read and approved the final manuscript.

## Conflict of Interest

ZZ, HY, and AT have filed patents related to this work. The remaining authors declare that the research was conducted in the absence of any commercial or financial relationships that could be construed as a potential conflict of interest.
