# Supplementary material for: MEK Inhibitor Augments Antitumor Activity of B7-H3-Redirected Bispecific Antibody
Source: Front Oncol. 2020 Aug 25;10:1527. doi: 10.3389/fonc.2020.01527 (PMC7477310; doi:10.3389/fonc.2020.01527)
Supplement: Supplementary file 1 [file Data_Sheet_1.docx]

Supplementary Material

## Supplementary Figures


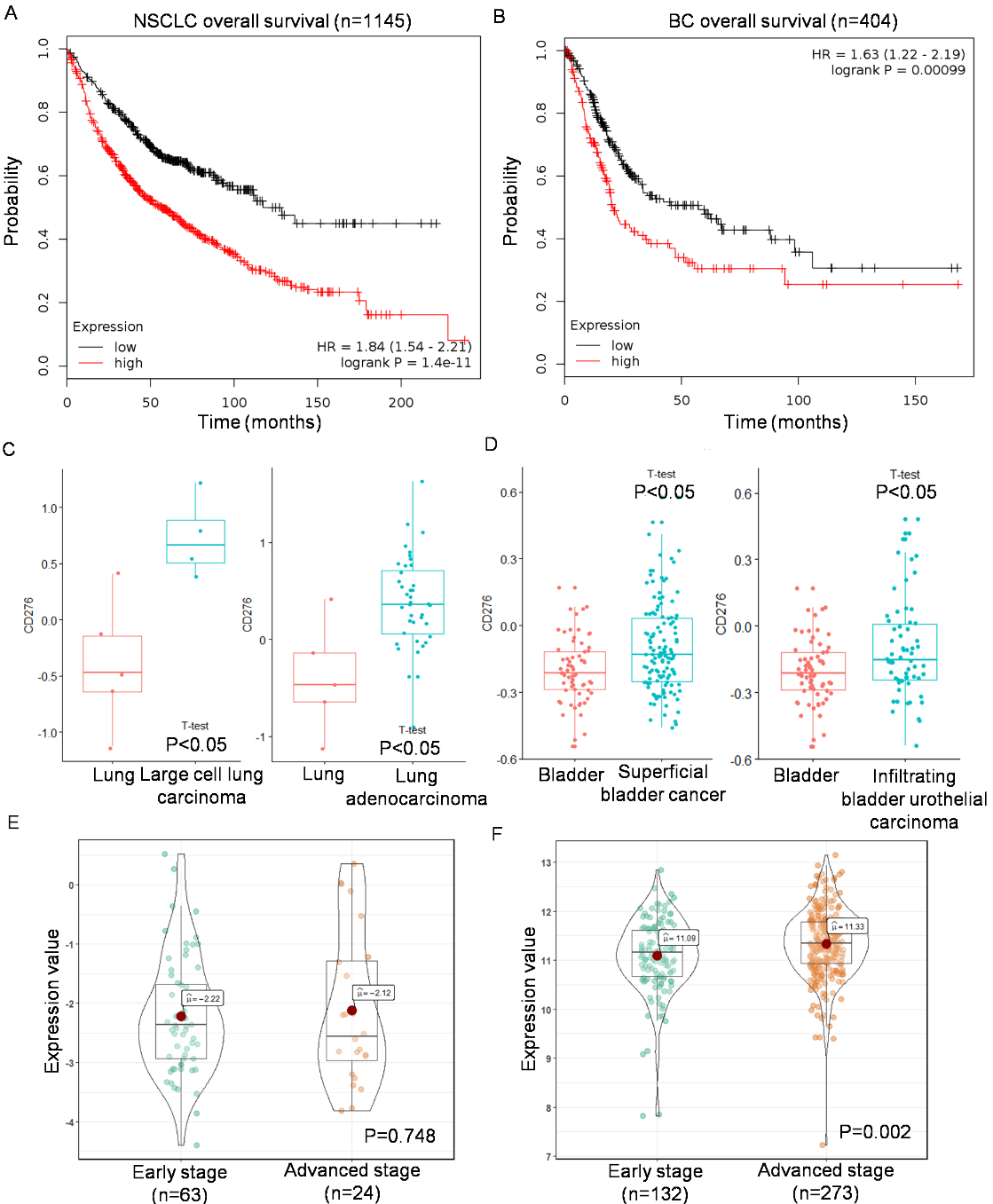


**Supplementary Figure 1** Analysis of B7-H3 expression and survival in NSCLC and BC. **(A, B)** Overall survival curves based on B7-H3 expression in NSCLC and BC patients (KM Plotter database). **(C, D)** Box plots derived from expression data comparing expression of B7-H3 in normal and cancer subtypes (Oncomine database). The analysis was shown in NSCLC subtypes relative to normal lung **(C)** and in BC subtypes relative to normal bladder **(D)**. **(E, F)** Comparison of B7-H3 expression among NSCLC **(E)** (Oncomine database) and BC **(F)** (TCGA database) patients at different clinical stages.


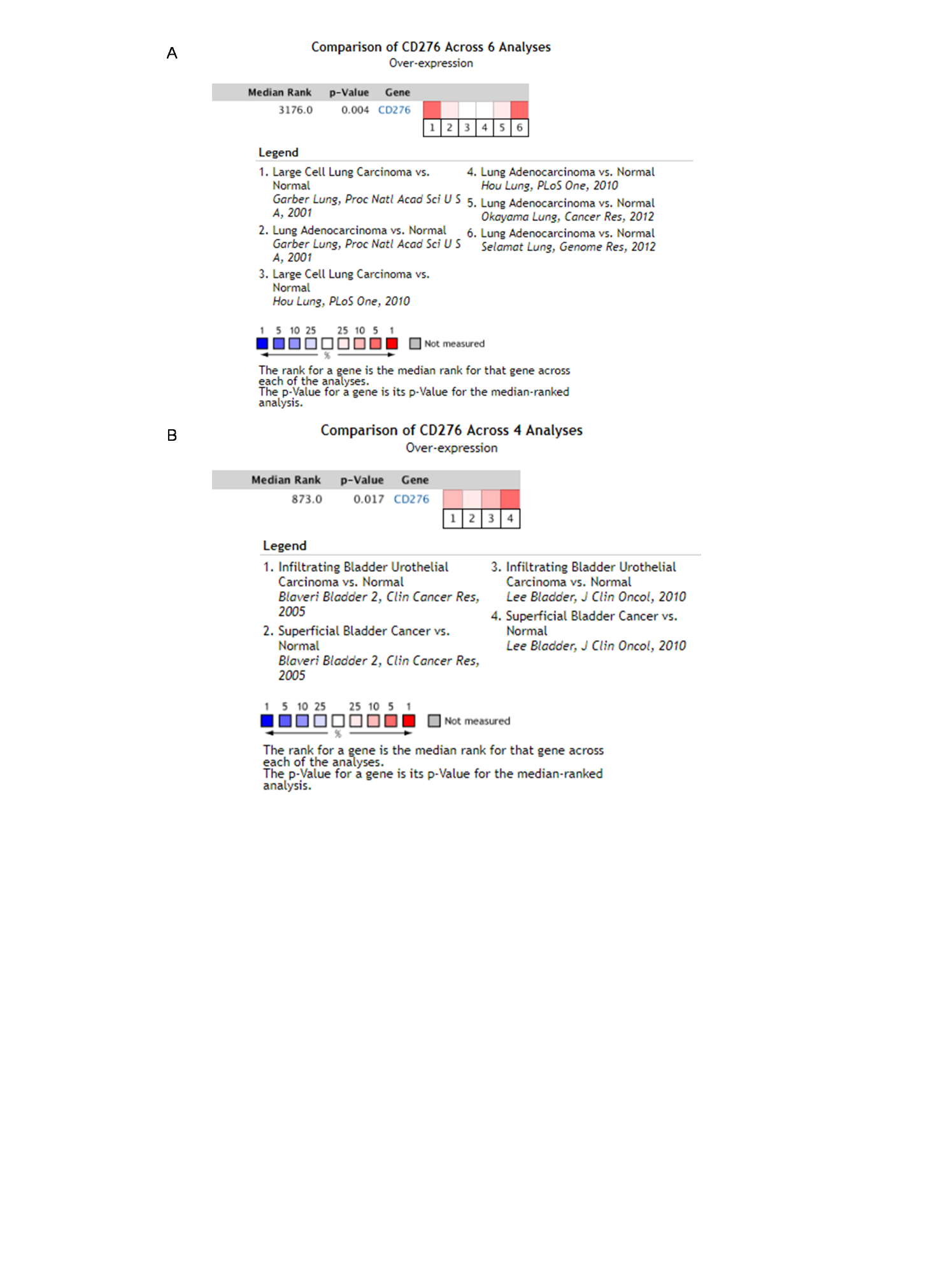


**Supplementary Figure 2.** Heat maps of B7-H3 expression level in NSCLC and BC derived from Oncomine database. **(A, B)** The ten studies showed the differential expression of B7-H3 in NSCLC and BC tissues compared with normal tissues. 1-6 in (A) and 1-4 in (B) represented NSCLC and BC, respectively. Darker red indicated higher B7-H3 expression in the chips.


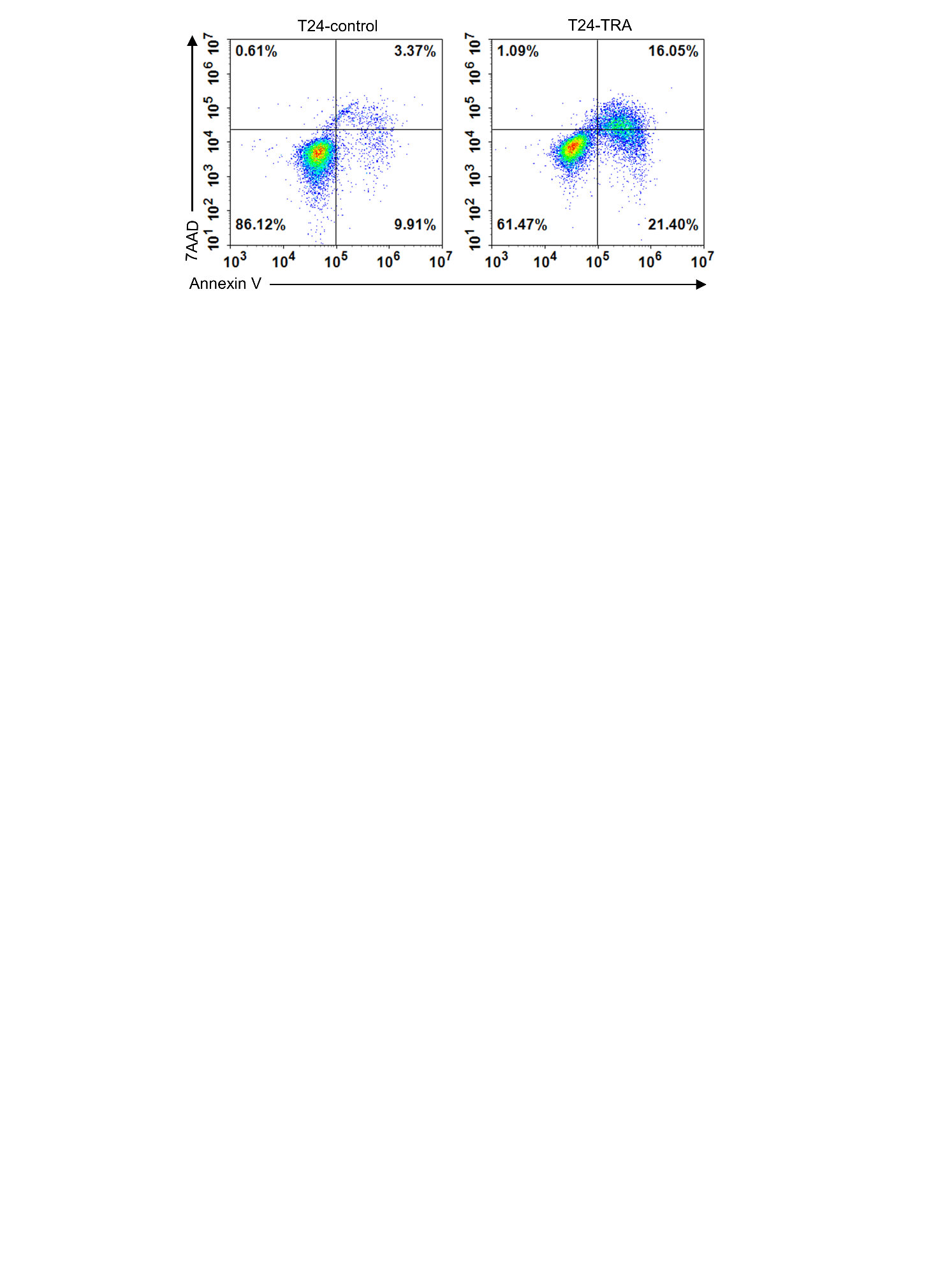


**Supplementary Figure 3.** Apoptosis detection with annexin V-FITC/7AAD double staining by flow cytometry. T24 cells were cultured with 10 μM trametinib for 48 hours.


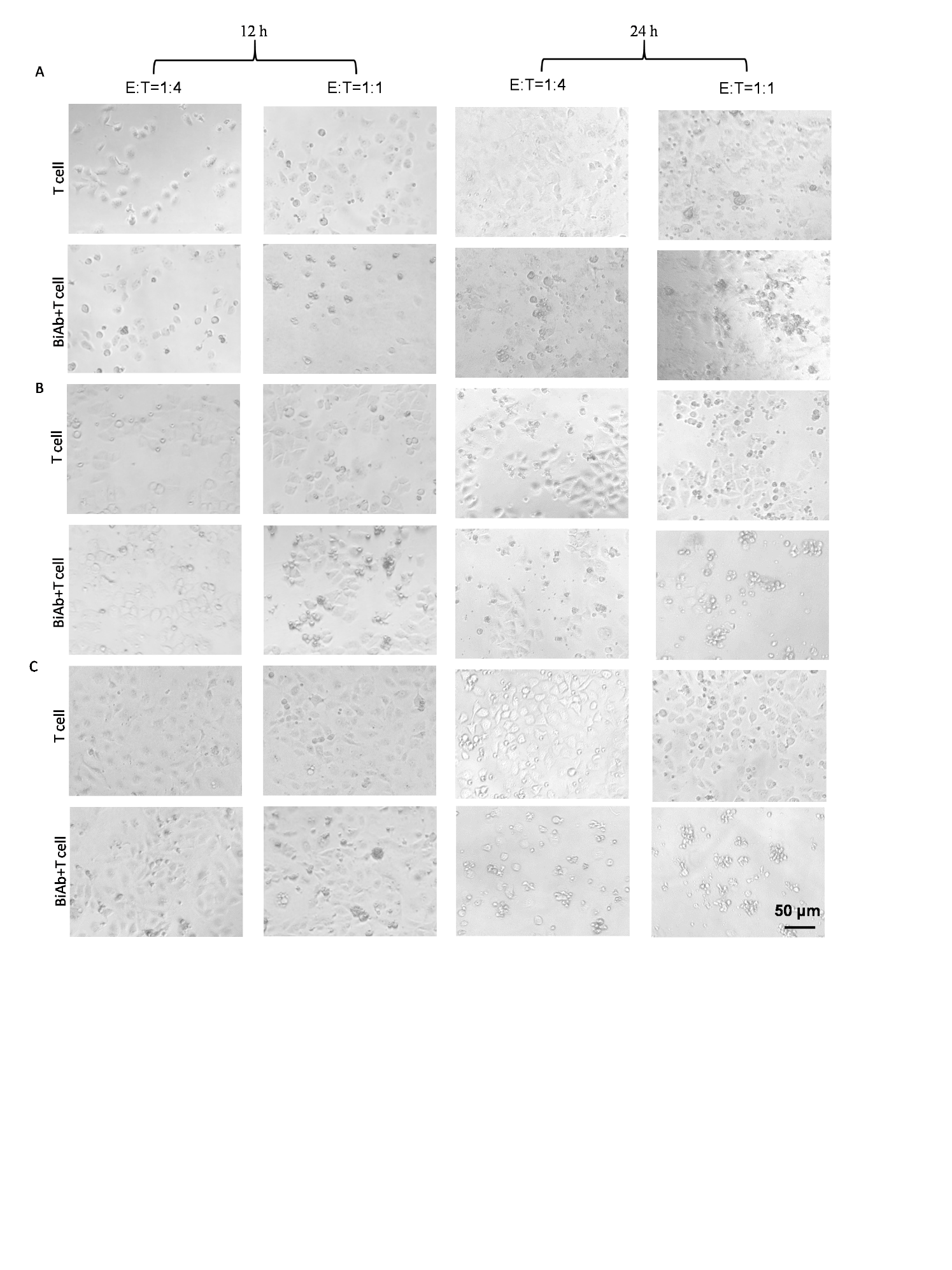


**Supplementary Figure 4.** Morphology of tumor cells after co-culture with human T cells. A549 **(A)**, H460 **(B)** or T24 **(C)** cells were co-cultured with T cells for 12 or 24 hours at a ratio of E:T=1:1 or 1:4. The BiAb group was treated with B7-H3×CD3 BiAb at a concentration of 5 μg/mL. Scale bar, 50 μm.


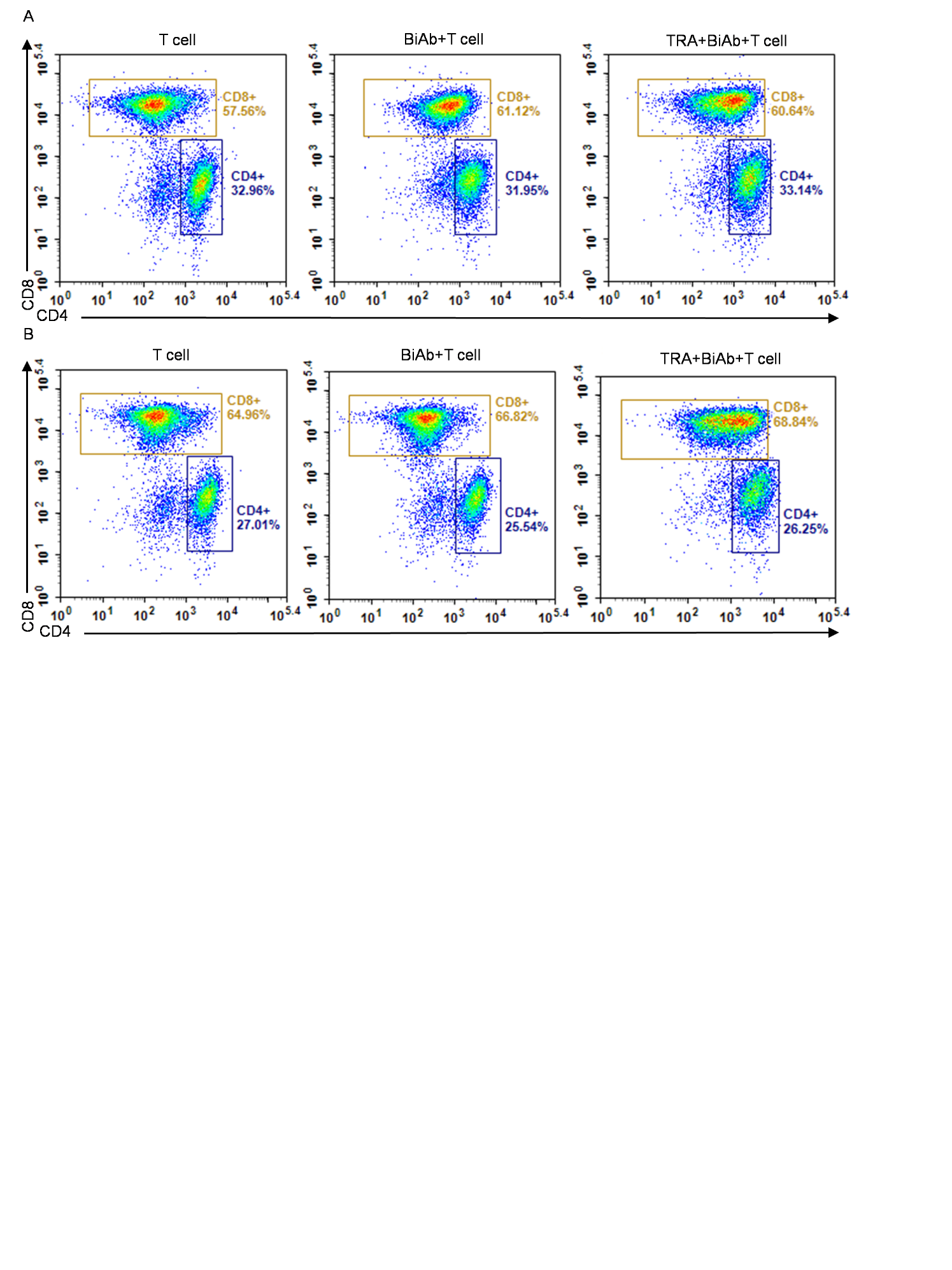


**Supplementary Figure 5.** The ratio of CD4 and CD8 positive T cells in coculture assay with tumor cells. **(A, B)** Dot plot diagram of flow cytometry showing CD4+ and CD8+ percentage of human T cells after coculture with H460 **(A)** or T24 **(B)** cells for 24 h in the presence of 1 μM trametinib alone or in combination with 5 μg/mL B7-H3×CD3 BiAb.

**A comparison between 8H9 and the antibody derived from our group**

8H9 is an B7-H3-targeted monoclonal antibody which has entered clinic trials for the treatment of various cancers (ClinicalTrials.gov: NCT00582608 and NCT01099644). Previously, Mahiuddin et al. tested the binding affinity of 8H9 in a study (1). For the antibody generated by our group, we have also examined the binding affinity with the same method (2). A comparison of the data suggested that our antibody exhibited a lower K_D_ value, which indicated a higher binding affinity. For antibodies, different affinity or antigen binding epitopes may have different antitumor effects, such as PD-1 antibody keytruda and opdivo.

1. Ahmed M, Cheng M, Zhao Q, Goldgur Y, Cheal S M, Guo H F, et al. Humanized Affinity-matured Monoclonal Antibody 8H9 Has Potent Antitumor Activity and Binds to FG Loop of Tumor Antigen B7-H3. J Biol Chem (2015) 290: 30018-29. doi: 10.1074/jbc.M115.679852.

2. Zhang Z, Jiang C, Liu Z, Yang M, Tang X, Wang Y, et al. B7-H3-Targeted CAR-T Cells Exhibit Potent Antitumor Effects on Hematologic and Solid Tumors. Mol Ther Oncolytics (2020) 17: 180-189. doi: 10.1016/j.omto.2020.03.019.
